# Plasma total anti-oxidant capacity correlates inversely with the extent of acute appendicitis: a case control study

**DOI:** 10.1186/1749-7922-1-6

**Published:** 2006-03-24

**Authors:** Mehmet Ozdogan, Ali Onder Devay, Ahmet Gurer, Eren Ersoy, Seda Duygulu Devay, Hakan Kulacoglu, Haldun Gundogdu

**Affiliations:** 1 Ataturk Teaching and Research Hospital, Department of General Surgery, Ankara, Turkey; 2 Gazi University Medical School, Department of Biochemistry, Ankara, Turkey

## Abstract

**Background:**

The role of free oxygen radicals in inflammatory conditions is well known. Free radicals cause lipid peroxidation of cellular membranes resulting in cell death. The purpose of this study was to investigate the levels of total anti-oxidant status (TAS), as a marker of anti-oxidant defense system and malondialdehyde (MDA), as a marker of oxidative stress, in the plasma of patients with acute appendicitis.

**Methods:**

Fifty-one adult patients with a median age of 31 years who underwent operations with a preoperative diagnosis of acute appendicitis were included in this prospective study. Blood samples for C-reactive protein (CRP), MDA and TAS were collected preoperatively. Groups were compared by using the Mann-Whitney *U *test.

**Results:**

There were 27 patients with acute phlagmenous appendicitis and 19 patients with advanced appendicitis (10 gangrenous and 9 perforated appendicitis), while 5 negative explorations were documented. No significant differences in WBC counts and MDA levels between groups were encountered. Plasma CRP was significantly higher in patients with perforated appendicitis, but not in the other groups. In advanced appendicitis group, TAS level was significantly lower than the other groups. On the other hand, plasma TAS level in acute phlagmenous appendicitis group was significantly higher.

**Conclusion:**

A decrease in plasma total anti-oxidant capacity might be a predictor of the progression of inflammation to the perforation in acute appendicitis.

## Background

Progression of the acute appendicitis (AA) following the initiation of the inflammation to the perforation has been investigated regarding pathological [1] and inflammatory parameters [2-7]. The role of free oxygen radicals (FOR) in various inflammatory conditions is well known. Free radicals, produced by polymorphonuclear leukocytes, cause lipid peroxidation of cellular membranes resulting in increased microvascular permeability, interstitial edema, inflammatory cell infiltration, neutrophil activation, and eventually cell death. The association between AA and oxidative stress has been addressed in a few experimental [8] and clinical [9,10] studies.

This study was conducted to reveal the levels of total anti-oxidant status (TAS), as a marker of anti-oxidant defense system and malondialdehyde (MDA), as a marker of oxidative stress, in the plasma of patients with AA.

### Patients and methods

Fifty-one adult patients (22 females, 29 males) with a median age of 31 years (15–71) who underwent operations with a preoperative diagnosis of AA at our center between January and March 2005 were included in this prospective study. Blood samples for C-reactive protein (CRP), MDA and TAS were collected preoperatively together with routine hematological and biochemical tests. The plasma was separated after centrifugation, and kept at -80°C for TAS and MDA levels measurements.

Plasma MDA levels were measured as described previously [11]. The total anti-oxidant status of the plasma was measured by using a Randox kit (Randox Laboratories, UK) with a novel automated colorimetric measurement method for total antioxidant response [12]. In this method, the hydroxyl radical, the most potent biological radical, is produced by the Fenton reaction, and reacts with the colorless substrate O-dianisidine to produce the dianisyl radical, which is bright yellowish-brown in colour. Upon the addition of a plasma sample, the oxidative reactions initiated by the hydroxyl radicals present in the reaction mix are suppressed by the antioxidant components of the plasma, preventing the colour change and thereby providing an effective measure of the total antioxidant capacity of the plasma. The assay results are expressed as mmol Trolox eq/L.

All appendectomy specimens were examined histologically by a pathologist who was unaware of the clinical diagnosis. The specimens were categorized as follows: *1) acute phlegmonous appendicitis (APA): *granulocyte infiltration of the appendiceal wall without any evidence of necrosis or perforation; *2) gangrenous appendicitis (GA): *with appendiceal wall necrosis; *3) perforated appendicitis (PA): *with rupture of the appendiceal wall to the serosal surface or with presence of visible perforation or localized abscess at the operation; and *4) negative exploration (NE): *normal appendix vermiformis without gross or microscopic inflammation and without any other intraabdominal inflammatory process.

Data were expressed as mean ± standard error of mean (SEM). Groups were compared by using the Mann-Whitney *U *test.

## Results

There were 27 patients with APA and 19 patients with advanced appendicitis (10 gangrenous and 9 perforated appendicitis), while 5 negative explorations were documented.

**Figure 1:**
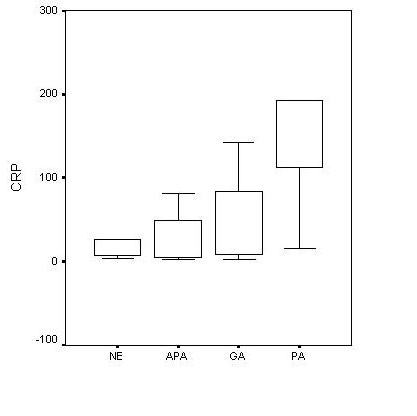
Mean values of serum CRP according to the different histological diagnosis.

No significant differences in WBC counts and MDA levels between groups were encountered (Table 1). Plasma CRP was significantly higher in PA group compared to the NE, APA and GA groups (Table 1 and Figure 1). The differences in CRP levels between NE, APA and GA groups were not statistically significant.

**Table 1:** WBC counts, and levels of CRP, MDA and TAS in groups.

	**WBC (cells/mm3)**	**CRP (mg/L)**	**MDA (nmol/ml)**	**TAS (mmol-Trolox-eq/L)**
**NE**	14.648 ± 2340	38.71 ± 25.88	4.24 ± 0.55	73.04 ± 0.71^#^
**APA**	12.831 ± 771	43.51 ± 11.47	4.32 ± 0.37	83.00 ± 0.92^¶^
**GA**	15.666 ± 1339	51.82 ± 21.43	4.63 ± 0.50	56.12 ± 1.23
**PA**	15.700 ± 1673	154.25 ± 20.97*	5.14 ± 0.32	59.04 ± 0.72

* *P *< 0.05 versus NE, APA and PA groups

^# ^*P *= 0.008 versus NE and advanced appendicitis group (PA and GA groups)

^¶ ^*P *= 0.009 versus APA and advanced appendicitis group (PA and GA groups)

**Figure 2:**
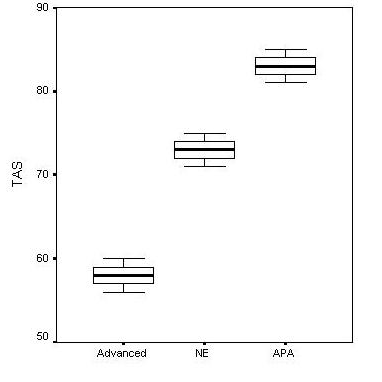
Mean values of serum TAS according to the different histological diagnosis (Advanced: PA and GA groups).

In regard of TAS levels, PA and GA groups displayed no significant differences. When these two groups were considered together as an "advanced appendicitis" group, TAS level was significantly lower than both NE and APA groups. On the other hand, plasma TAS level in APA group was significantly higher than that of the NE group (Table 1 and Figure 2).

## Discussion

Oxidative stress can be defined as an imbalance between oxidants and antioxidants, an excess of oxidants and/or a depletion of antioxidants, in the organism. Lipid peroxidation in cellular membrane, which is caused by FOR, leads to MDA release, and this indicates the degree of oxidative stress and tissue injury [13]. Plasma TAS level is another well-established marker of oxidative stress, indicating the anti-oxidant defense status of the organism [14].

C-reactive protein, as a non-specific inflammatory acute phase reactant, has been considered to be an independent predictor of AA [4,6]. However, its value is controversial in the diagnosis of AA, and WBC count was found to be superior to CRP in some studies [3,7]. In general, CRP values are predictive of inflammation severity. Previous data have shown that the greater the degree of appendicular inflammation the greater the CRP value, reaching maximum values in cases of perforation [2]. Thus, CRP has been suggested to be useful especially in discriminating the perforated appendicitis cases [4,7] and [7]. Similarly, the present study has revealed a significant increase in CRP in appendicitis progressed to perforation.

In a study investigating the role of FOR in experimental appendiceal inflammation, it was found that the serum catalase and glutathione peroxidase activities were increased following the ligation of the appendix [8]. Authors suggested that FOR increased sufficiently to activate the enzymatic defense system in acute appendicitis. In a clinical study, it was demonstrated that thiobarbituric acid reactive substance, an oxidative stress parameter, and superoxide dismutase activity were higher in gangrenous appendicitis than less advanced appendicitis [10]. Similarly, in pediatric appendicitis patients it was found that elevated levels of MDA and superoxide dismutase activity were present in more advanced appendicitis [9]. These cumulative data indicate that FOR gradually released during the progression of the inflammation may induce an increase in the anti-oxidant defense capacity of the organism.

There are some controversies between our findings and these data. The serum levels of MDA were not different between groups in the present study. Nevertheless, TAS activities were significantly different between groups. Plasma from patients with advanced appendicitis (i.e GA and PA groups) presented significantly reduced TAS activity in comprison with APA group and negative exploration cases. It is known that patients with sepsis and systemic inflammatory response syndrome had a more severe oxidative stress and lower plasma total antioxidant capacity than patients without this syndrome [15,16]. The findings of the present study may indicate that an induced anti-oxidant activity during the initial phases of inflammation might be followed by a reduction in total anti-oxidant capacity as the severity of the inflammation progresses. This reciprocal relation may indicate an endogenous deficiency of the anti-oxidant systems resulted from consumption in the presence of more severe inflammation, or vice versa, lower total anti-oxidant capacity may result in progression of inflammation to gangrene and eventually perforation.

On the other hand, TAS level of APA group showed no difference comparing NE group. We should keep one point in mind that NE group already had an obviously increased CRP level. Tough no intra-abdominal, including gynecological, pathology was found in intra-operative exploration, the patients in this group might have an inflammatory state in another part of the body. Thus, we could say that acute appendicitis in its initial phase does not display a specific TAS value than a nonspecific inflammation and it does not seem to be possible to use TAS value as a diagnostic marker in diagnosis of acute appendicitis.

## Conclusion

Although plasma total anti-oxidant capacity can not be used in the diagnosis of acute appendicitis, a decrease in its value by time might be consider as a predictor of the progression of inflammation to the perforation in acute appendicitis cases.

## Competing interests

The author(s) declare that they have no competing interests.

## Authors' contributions

MO: Conception and design, analysis and interpretation of data, drafting the manuscript

AOD: Acquisition of data, conception and design

AG: Conception and design, acquisition of data

EE: Conception and design, acquisition of data

SDD: Acquisition of data

HK: Analysis and interpretation of data, revising it critically

HG: Revising it critically, final approval of the version to be published

All authors read and approved the final manuscript.
